# A homology-based pipeline for global prediction of post-translational modification sites

**DOI:** 10.1038/srep25801

**Published:** 2016-05-13

**Authors:** Xiang Chen, Shao-Ping Shi, Hao-Dong Xu, Sheng-Bao Suo, Jian-Ding Qiu

**Affiliations:** 1 Department of Chemistry, Nanchang University, Nanchang 330031, P.R. China; 2 Department of Mathematics, Nanchang University, Nanchang 330031, P.R. China; 3 Department of Materials and Chemical Engineering, Pingxiang University, Pingxiang 337055, P.R. China

## Abstract

The pathways of protein post-translational modifications (PTMs) have been shown to play particularly important roles for almost any biological process. Identification of PTM substrates along with information on the exact sites is fundamental for fully understanding or controlling biological processes. Alternative computational strategies would help to annotate PTMs in a high-throughput manner. Traditional algorithms are suited for identifying the common organisms and tissues that have a complete PTM atlas or extensive experimental data. While annotation of rare PTMs in most organisms is a clear challenge. In this work, to this end we have developed a novel homology-based pipeline named PTMProber that allows identification of potential modification sites for most of the proteomes lacking PTMs data. Cross-promotion E-value (CPE) as stringent benchmark has been used in our pipeline to evaluate homology to known modification sites. Independent-validation tests show that PTMProber achieves over 58.8% recall with high precision by CPE benchmark. Comparisons with other machine-learning tools show that PTMProber pipeline performs better on general predictions. In addition, we developed a web-based tool to integrate this pipeline at http://bioinfo.ncu.edu.cn/PTMProber/index.aspx. In addition to pre-constructed prediction models of PTM, the website provides an extensional functionality to allow users to customize models.

With many organism genomes being sequenced at an ever-accelerating pace, a vital and challenging issue is inferring protein functions and their downstream regulatory signals^1^. Mostly regulatory signals are transmitted by alteration of translated proteins through the covalent addition of functional groups or proteins of post-translational modifications (PTMs) to key residues to increase their functional diversity^2^. These signals are then relayed and amplified which influences almost all aspects of normal cell biology and pathogenesis. Therefore, identifying and understanding a cellular PTM site atlas is critical, which in basic research of cell biology and disease treatment and prevention has many applications^3^.

As experimental strategies for identification of PTM sites is costly and time-consuming, *in silico* identification of PTM sites provides a potentially alternative strategy for query proteome/organism (for which PTM data is unavailable) annotation with a low cost^4^. As a result, for a query dataset the best prediction always performs from the model trained using data from the same organisms^5^,^6^, while available algorithms are often data-depended that model construction and model prediction would use the same organisms or tissues data. Although dozens of algorithms are now available^7^ and online resources contain a wide range of knowledge about various PTMs for common organisms such as human, bovine and mouse, yet PTM annotation for most of organisms or tissues lacking experimental PTM data is challenging which has become a bottleneck limiting the contribution of PTM study to biological understanding. To address this limitation, one feasible approach is to use existing experimental PTM data of known proteome as the starting point for annotation of a new query organism or query proteome.

Here, we introduce PTMProber (PTM Prober), a homology-based pipeline dedicated to the deciphering of PTM sites atlas for all query proteomes, particularly for the organisms and tissues for which PTM site data is unavailable. Using query proteome of *Ectocarpus siliculosus* (brown alga, taxonomy ID = 2880) as case, taking advantage of sequence homology between *Ectocarpus siliculosus* and the organism proteome for which experimental PTM data is available (referred to as known organism or known proteome), this pipeline would automatically use query peptides of *Ectocarpus siliculosus* that contain putative PTM sites as BLAST (Basic Local Alignment Search Tool)^8^ queries and align PTM sites of known proteomes to identify the putative sites in *Ectocarpus siliculosus*. If a query in the proteome of *Ectocarpus siliculosus* and its best match in known organisms had few or no sequence differences, the query would be considered a true putative PTM site from *Ectocarpus siliculosus*. Note that BLAST^9^ as the most popular similarity search tool are used to determine the similarity degree of sequence between PTM sites in known organisms and those in query organisms. Based on well-established reciprocal BLAST hits algorithm^10^, the stringent benchmark of cross-promotion e-value (CPE) as the algorithmic core used for PTMProber pipeline to identify homologous PTM sites. Moreover, the databases of experimental PTM data and query proteome data used for BLAST searches in PTMProber pipeline that is considered data core. Reliable experimental PTM data in multiple PTM types are collected and utilized to construct BLAST database using *makeblastdb* program^9^. All of detailed algorithms are presented in the methodology section.

PTMProber pipeline has been implemented as a web-based tool that can be easily used to perform large-scale PTM prediction for query proteomes in an automated fashion. Applications of PTMProber pipeline on three organism proteomes yielded over 58.8% homologous PTM sites with over 70% precision. Comparison tests with other machine-learning tools showed that PTMProber pipeline performs better on general predictions. In PTMProber V1.0, we have collected PTM data from several sources^11^,^12^, and then constructed general prediction models for five PTM types including phosphorylation (serine, threonine and tyrosine), acetylation (lysine), ubiquitination (lysine), methylation (lysine and arginine), sumoylation (lysine) that were used for cellular conditions of 340 organisms. It is noted, however, that using the current pre-constructed models users cannot correlate prediction with other particular PTM; the cellular conditions of query proteome come not from the 340 organisms. To this end, PTMProber V1.0 has a novel functionality to construct BLAST databases utilizing user data for rapidly customizing condition-specific prediction model. Especially for PTM data in biological events or pathway processes, this approach can be used to investigate the conservation of biological responses across species or conditions^13^. In addition to pipeline extensibility it encompasses features allowing (i) batch prediction in both ID entry and file upload modes, which makes large-scale predictions become an effortless task for users; (ii) better identification of query proteomes as compared to traditional machine-learning based predictions for which PTM data in proteome is unavailable; (iii) multi-parameter adjustment for users that are interested in the stringency control of predictions; (iv) rapid identification for one query proteome in a few hours; (v) simultaneous multisite predictions for multi-residue PTMs; and (vi) email delivering and HTML storage of prediction results after submission. Finally, PTMProber is freely available at http://bioinfo.ncu.edu.cn/PTMProber/index.aspx that provides a valuable pipeline for biologists to predict PTM sites up to the whole proteome level.

## Results

### Test PTMProber pipeline by reconstructing model

PTMProber is a homology-based pipeline that is first of its kind to identify PTM sites. It is difficult to evaluate our pipeline by comparing with existing tools. Therefore, model-reconstruction approach is designed to validate the performance of pipeline. Based on the descriptions in the section of Performance Evaluation, we have reconstructed two models (referred to as M1 and M2, M1 represents PTMProber M1 model that constructed by excluding PTM data in assigned test organism from all collected PTM data. M2 represents PTMProber M2 model that constructed by all collected PTM data.) for each type of PTMs including phosphorylation, acetylation, ubiquitination, methylation and sumoylation on *Rattus norvegicus*, *Gallus gallus* and *Bos taurus*. PTM data for five PTM types in three organisms respectively would be used as testing. The testing data collected from PhosphoSitePlus/UniProt database is listed in Supplementary Table 1. The results show that the pipeline can accurately predict known PTM sites with over 58% recall in PTMProber M1 and 100% in PTMProber M2 for different PTMs on the three organisms. Moreover, our pipeline can identify PTM sites with a high precision for different PTMs (see Table 1). Note that the PTM state is determined through CPE benchmark in above results.

### Compare performance between E-value and CPE

Although the E-value method represents a general benchmark for assessing the homology, this benchmark suffers from drawbacks for predicting PTM sites. Due to limitations in window length of query sequence, the associated query protein corresponding to the best match may not be homologous to a known protein corresponding to the known sequence. Overbeek *et al*.^10^ proposed reciprocal BLAST hits method to identify homologous genes. Here, our pipeline refers this well-established strategy to create CPE benchmark for ascertaining homologous PTM sites. In order to compare CPE with E-value benchmark and evaluate the performance which ascertains PTM sites, we adopt the PTMProber M1 model to identify the above testing data (see Supplementary Table 1) by using CPE and E-value benchmark separately (BLAST parameters were default for each PTM). The test results for different PTM types were compared as shown in Supplementary Table 2. Note that the percentages of predicted PTM sites in identical sequence differences are similar between the two benchmarks. These results also indicate that the CPE benchmark less sensitive but more specific than E-value benchmark. A sample example can illustrate this. The site “S459” in query protein P06536 (serine in position 459 of protein) and its the best match phosphorylation site “S578” from *Homo sapiens* with accession number P10275 has been identified by E-value benchmark. Despite of identical local sequence, these two proteins (P06536 and P10275) are not homologous by CPE benchmark. Also, there was no evidence to indicate that the site “S459” in query protein P06536 can be phosphorylated by any kinase^14^.

### Method Comparison

**Figure 1:**
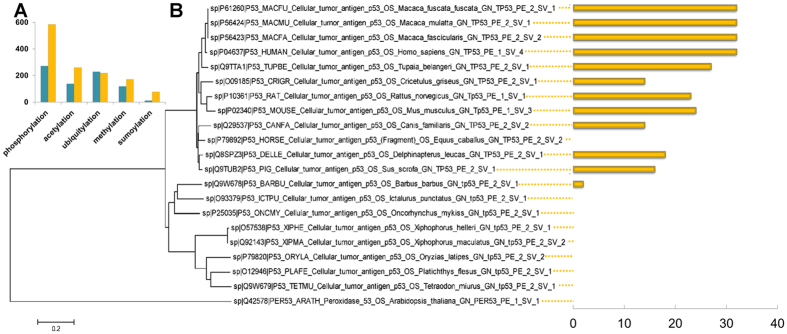
Method comparison. (**A**) Comparing the number of predicted PTM sites (*y*-axis) in p53 family proteins between MLA (machine-learning approach, yellow bars) and SSA (sequence similarity-based approach for PTMProber, peacock blue bars) for five PTM types. Our five MLAs works include SubPhosPred tool for phosphorylation, PSKAcePred tool for acetylation, UbiProber tool for ubiquitination, PMeS tool for methylation and SUMOAMVR for sumoylation. (**B**) Distribution of the number of phosphorylation sites (*x*-axis) predicted on the 21 proteins of p53 family by PTMProber.

*In silico* identification of post-translational modifications is potentially a useful alternative strategy for whole proteome annotation, which has always two categories of strategy. Various machine-learning approaches (MLAs) including Support Vector Machine^15^, Random Forest^16^ and Bayes^17^ fall into the first category. As a result, many studies have utilized these approaches to create software tools for predicting PTM sites^18^,^19^,^20^, and also we had developed some tools for identification of various PTM based on MLA. The latter category is sequence similarity-based approaches (SSAs) that include BLAST^8^, PSI-BLAST^21^ and Motif-x^22^. In this work, a pipeline called PTMProber was developed that utilizes sequence homology to construct models for identifying putative PTM sites in a query proteome of interest. It has the advantage of more accurate prediction of post-translational modification with lower false-positive rate when compared to machine-learning methods. For this a very important reason is that it considers the similarity for a complete protein that encompasses different features, while traditional MLAs only consider local sequence information. Here, we took 21 proteins corresponding to different organisms belonging to the p53 family that were collected as case study to compare PTMProber pipeline and MLAs tools developed by us^23^,^24^,^25^,^26^ on phosphorylation, acetylation, ubiquitination, methylation and sumoylation. In fact, the results of case study are indicated in Fig. 1 that nearly agrees with what we had already said. As can be seen from Fig. 1A, the latter obtain more candidates than the former that achieved 100% precision. While for the latter the majority of the candidates in the 21 proteins have not been annotated in current database. This provides a reasonable judgment for our pipeline that could reduce false-positive rate. Two examples from test results are shown with DOG^27^ in Supplementary Fig. 1 to demonstrate that the former precisely obtain almost all verified sites for five PTM types. In addition, the distantly evolved proteins have absolute difference in sequences and structures. As shown in Fig. 1B, phosphorylation predictions for the p53 family in our pipeline also indicate that more PTM sites can be reproduced for closely evolved organisms. Note that PTMProber precisely returns all verified phosphorylation sites in online database for this prediction.

### PTMProber Extensibility

**Figure 2:**
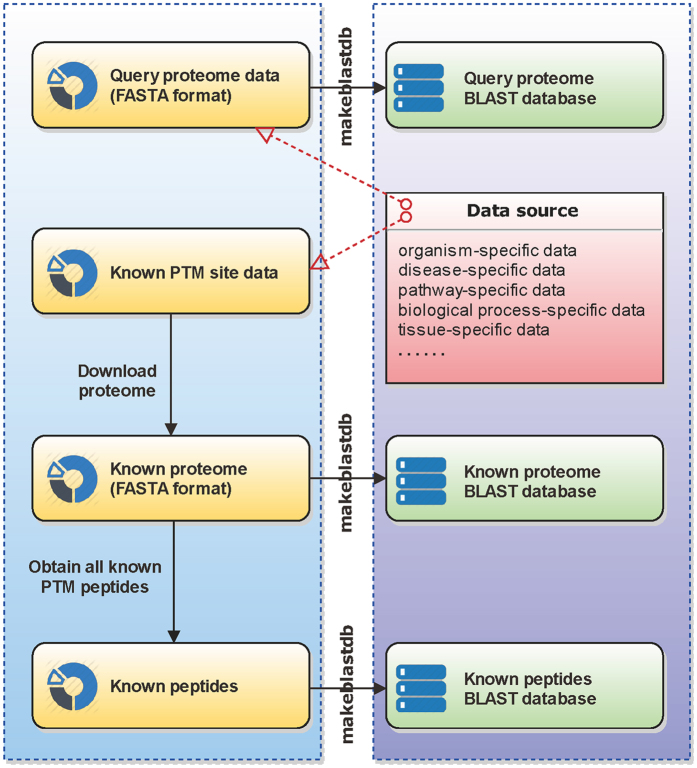
Extensible contents of PTMProber. The extensible source (yellow box) includes data of known proteome that is constructed known proteome BLAST database and known peptides BLAST database and data of query proteome that yields query proteome BLAST database. PTMProber pipeline runs PTM prediction by invoking these databases (green box) updating from various data source (red box). The databases generated from makeblastdb programs in the blast-2.2.28+ package.

PTMProber V1.0 functionality currently comprises PTM predictions for phosphorylation (serine, threonine and tyrosine), acetylation (lysine), ubiquitination (lysine), methylation (lysine and arginine), sumoylation (lysine) used for 340 organisms. As PTM data in various organisms, tissues, even specific diseases, pathways, and biological processes is accumulating rapidly, and as the data of cellular conditions is ever-changing, it is difficult for a prediction server to keep track of all available data of PTM or cellular conditions. Although the V1.0 contains mainly several PTMs and serves 340 different organisms, it’s still far from being adequate for certain users. To perform proteome-specific scale prediction of PTM sites in an automated fashion, PTMProber V1.0 provides an extensible utility that enables users to customize their own models using condition-specific PTM data. To customize a predictable model of PTM under the assigned conditions, users only need to update the aligning databases. As can be seen from Fig. 2, there are three aligning databases can be replaced including protein database of known proteome, its associated known peptide (in the center of peptide segment contained a PTM site) database, and query proteome database (protein used as a query identification in our pipeline must exist in query proteome). These databases generate by using specific parameters and performing three makeblastdb programs. The users must provide own data file in FASTA format for constructing each aligning database. Although keeping simplicity, the generating files of makeblastdb program must be included the files suffixed phr, pin, psd, psi, psq, pnd and pni, or it will fail to further extract data from aligning databases with blastdbcmd program in the customized task. If a user just wants to add new PTM types to PTMProber pipeline or increase the number of known PTM data to current models, known proteome database and associated known peptide database need to be updated. An updating of query proteome database can identify PTM sites in more organisms or proteome. In the following, we will include more PTMs and extend this pipeline to various specific conditions for experimental PTM studies.

### Web System development

The web tool PTMProber follows HTML5 and CSS3 strict in W3C standard to ensure consistency of display view across different browsers. Asynchronous JavaScript technology is utilized to control the submissions of web form and dynamically display the status of the predicting process. With the Simple Mail Transfer Protocol (SMTP) of WebMail object in ASP.NET the prediction results can easily send to user-provided email address from PTMProber server without waiting after user submission. PTMProber took average 5 seconds to predict for a protein in a PTM type performed by a machine with a 3.1 GHz processor and 2 GB of memory.

The PTMProber website was constructed in an easy-to-use manner. The primary entry point to PTMProber is its introduction interface, and clicking search link entries the search interface that was shown in Supplementary Fig. 2A. This search interface contains six steps for various PTM predictions of query protein. There are three main input steps (step 1–3) for data uploading that include query protein of your prediction, its associated BLAST database of query proteome and the BLAST database of known proteome. All of those steps have two ways to upload the data, and the users can only choose a way for a prediction. For the parameters configuration, three options can be preset including the options of scoring matrix, expect value (E-value) for saving BLAST hits and the maximum number of aligned sequences. User-provided email address can be used to send the prediction results to this mailbox without waiting after submitting prediction form. In the controlling panel, the example button provides a presetting form to testing PTMProber for a new user.

As mentioned earlier, although we provided the models for five PTMs used for the cellular conditions of 340 organisms, it is difficult to cover all available data of PTM or cellular conditions. The web-based tool also provides pipeline extendibility. There are two toolkits involving customizing the BLAST database of query proteome and the BLAST database of known proteome that were integrated in this tool. We will describe the extensible usages of this web interface. For the former, the user must upload protein sequence file in FASTA format to server backstage, which will create a new BLAST database and return a unique name for the new database to the user in a few minutes (Supplementary Fig. 2B). This name can always be used as an alternative input in step 2 (see Supplementary Fig. 2A). For the latter, two files including the known proteome with FASTA format and its associated PTM annotations with one row per protein (every protein in known proteome is forced to match the protein associated annotations of PTM site exactly) will be uploaded simultaneously, when entering a window length (odd integer) and submitting to server backstage, which will produce two BLAST databases for the known proteome as well as its known peptides in the server backstage and return a unique name for two interconnected databases to the user (Supplementary Fig. 2C), and this also takes a few minutes only. It will be an alternative input in step 3 once and for all (see Supplementary Fig. 2A).

Finally, we have used an example presented in Supplement Text 1 to explain the various features of the search interface (see Supplementary Fig. 2D).

## Discussion

In fact, we found that more 90% of experimentally-verified PTM annotations only belong to three model organisms (*Homo sapiens*, *Mus musculus*, *Rattus norvegicus*) in UniProt database. Most of the organisms do not have any experimentally-verified PTM terms. With the increasing gap between available proteins and annotated PTM-proteins, it is clear that PTM annotation of all proteins can only be accomplished by combining experimental and computational approaches. Thus, the development of computational methods is critical for filling the gap between homologous organisms. The two important computing methods SSA and MLA provide the first evidence for PTM of a protein. While the homology-based SSA can improve precision for PTM in new homologous organisms that is more effective than data-depended MLA. Our pipeline employs SSA and integrates a novel CPE benchmark that allows identification of putative protein PTMs with a higher confidence.

Some putative PTM sites in query proteome originate homologous protein of known proteome that has highly reliability^13^. Homology-based PTMProber pipeline can be applied to any domain of research that requires knowledge of PTM targets, here we would like to highlight one application that is PTM identification in specific organism proteome, particularly for non-model organisms. This will greatly reduce the workload for hypothesis-driven experiments of PTMs in a novel proteome. PTMProber also can be used to protein/peptide array as one typical application that identify putative PTM sites in an interesting organism, and then design a protein/peptide microarray for studying cellular signaling in a high-throughput manner^28^,^29^. Moreover, PTMProber can bring experimental hypothesis into proteomics studies of PTMs. For example, it will prompt many researchers to develop PTM networks of novel organism that more accurately reproduce relevant aspects of human physiology^30^. From such investigations, it is clear that specific organisms and its organs or tissues often serve as more effective experiment objects of particular human diseases^31^,^32^. Note that the sites reported by PTMProber are only predictions; further, the functional significance of a homologous site may differ in the target organism, especially when the target is a distantly related organism. More realistically, customized model in PTMProber makes it possible for users, especially for the non-computational biologists, to train models using PTM data specific to their own work. Hence, PTMProber pipeline for proteome annotation and large-scale experimental design of PTM has a highly valuable as increasing dramatically of genome and proteome data.

## Materials and Methods

### Materials

**Figure 3:**
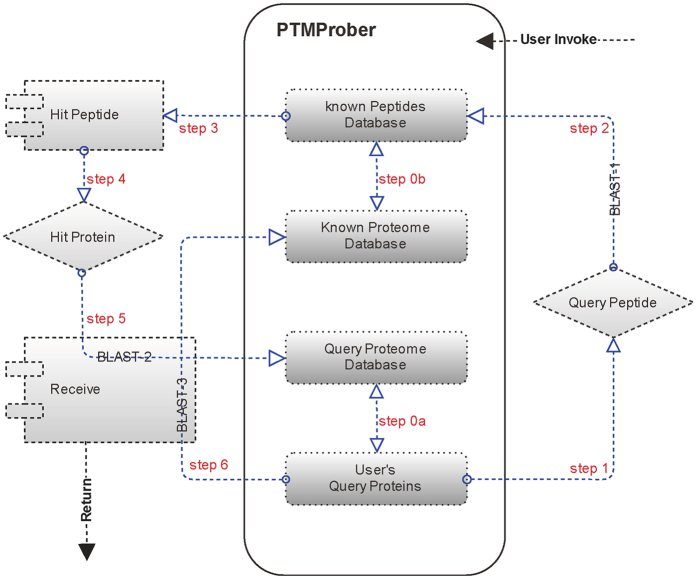
System flow of PTMProber pipeline. The gray box represents the computing node, while red characters correspond with the numbered steps in PTMProber pipeline. The step 0a represents that the protein as a query must correspond to query proteome database; and the query protein database must contain query protein. The step 0b acquires the known peptide from the known proteome, and vice versa. The steps 1–6 represent that search or obtain the corresponding symbol contained in gray box. The steps 5–6 (BLAST-2 and BLAST-3) ascertain whether user’s query proteins contain PTM sites.

PTM data for five pre-constructed models, phosphorylation, acetylation, ubiquitination, methylation, and sumoylation model, from several sources including UniProt/Swiss-Prot^11^, and PhosphoSitePlus^12^ (Jan. 8, 2015) were collected to construct known proteome database (see Fig. 3). Subsequently, by sliding a scaled window along each of the proteins, we extracted peptide segments with window length of 2*w* + 1 centered on a PTM residue, *w* residues upstream and *w* residues downstream of the PTM site. The window length was set according to different PTM types. Peptide segments with length less than the window length were complemented by blank character. Peptides with centered residue able to be formed PTM were regarded as known peptides database (Fig. 3). Supplementary Table 3 lists the PTM data sources and statistics for different PTM types.

For the first version of PTMProber we have collected proteome data of 340 organisms from UniProt/Swiss-Prot (Jan. 8, 2015) to construct query proteome database of the different cellular conditions. Similarly, the routing process of peptide segments was done to create query proteome database for each proteome query. Specific modification residues of PTM types were as centered site. The index of 340 organisms was listed in Supplementary Table 4.

### Methods

Sequence similarity searching is one of the most important bioinformatics activities and often provides the first evidence for the function of a newly expressed protein or piece of sequence, which is the same as gene finding^9^. Gene finding is one of the first and most important steps in understanding the genome of a species once it has been sequenced. Therefore, as the entire proteomes of many different species are characterized, a promising direction in current research on protein finding is a comparative proteomics approach. This also includes prediction of diverse functional groups such as PTMs in regulatory regions of protein. The Basic Local Alignment Search Tool (BLAST) is the most widely used sequence similarity tool^9^. Standard protein-protein BLAST (blastp) program^33^ is used for both identifying a query amino acid sequence and for finding similar sequences in protein databases. Like other BLAST programs, blastp is also designed to find local regions of similarity. When sequence similarity spans the whole sequence, blastp will also report a global alignment, which is the preferred result for protein finding purposes. Note that all related BLAST programs come from blast-2.2.28 + package^9^ in the following descriptions of methodology.

Using *Bos taurus* as a test organism, Jalal *et al*.^13^ previously proposed a concept that utilize blastp to determine homologous phosphorylation peptides for species of interest. Based on this concept we construct a global prediction pipeline of PTM sites called PTMProber, which invoke three blastp programs and four available components for input: user query protein and associated query proteome database and known proteome database as well as associated known peptides database (see Fig. 3). The first BLAST step (BLAST-1) is that query peptides obtained from query protein align known peptides database to determine the most match known peptide (refer as hit peptides, the corresponding protein refers as hit protein). The second BLAST step (BLAST-2) is to determine the smallest E-value (the E-value of best match) of hit protein obtained from the first step aligns query proteome database and the E-value of hit protein aligns the most match query protein respectively. The third BLAST step (BLAST-3) is respectively to determine the smallest E-value (the E-value of best match) of query protein aligns known proteome database and the E-value of query protein aligns hit protein. Note that query protein must be contained within query proteome database in the above steps, and this also why a query proteome database is necessary for our pipeline (Fig. 3). Finally, the information obtained from BLAST-2 and -3 is used to determine PTM state and return to user by the following formula


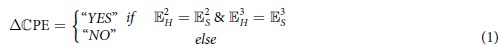


where 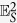 and 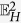 obtained by run BLAST-2 that are respectively the smallest E-value (the E-value of best match) and the E-value of hit protein aligns query protein (generated by using hit protein as BLAST query align query proteome database). Also 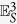 and 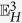 obtained by run BLAST-3 respectively are the smallest E-value (the E-value of best match) and the E-value of query protein aligns hit protein (generated by using query protein as BLAST query align known proteome database). Here, 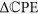 (cross-promotion e-value) is a stringent benchmark referred reciprocal BLAST hits method^10^ that employs to ascertain homology. Let 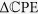 = “yes” if the E-values from BLAST alignment are reciprocal that both 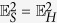 and 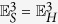 are met simultaneously, or 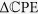 = “no”.

### Performance Evaluation

In order to evaluate the performance of identification PTM sites, two reconstructed models would be customized by creating BLAST databases for known proteome and its associated peptides (customized method see the section of Web system development). PTM data of an assigned organism excludes from PTM data of all known proteome that was used to construct one model. The other one was generated by using all PTM data of known proteome which includes the assigned organism. Finally, PTM data in an assigned organism will be used in this evaluation to test the models and exemplify our pipeline.

## Additional Information

**How to cite this article**: Chen, X. *et al*. A homology-based pipeline for global prediction of post-translational modification sites. *Sci. Rep*. **6**, 25801; doi: 10.1038/srep25801 (2016).

## Supplementary Material

Supplementary Information

## Figures and Tables

**Table 1:** Test results for PTMProber pipeline.

PTM	Rattus norvegicus (%)		Gallus gallus (%)		Bos taurus (%)	
	M1	M2	M1	M2	M1	M2
Phosphorylation	84.6/61.8	81.8/100.0	90.1/74.7	85.4/100.0	82.5/72.9	79.6/100.0
Acetylation	84.6/76.4	83.8/100.0	91.7/84.6	86.7/100.0	89.5/80.6	80.4/100.0
Ubiquitination	71.5/70.3	68.7/100.0	100.0/100.0	100.0/100.0	100.0/100.0	100.0/100.0
Sumoylation	95.4/72.4	93.5/100.0	75.0/75.0	70.9/100.0	83.3/80.0	71.4/100.0
Methylation	85.1/58.8	82.2/100.0	81.8/75.0	78.1/100.0	92.4/74.7	84.2/100.0

The segregative digits in each cell by backslash character represent the ratio of correct prediction sites in all prediction sites by model (refer to precision) and the ratio of correct prediction sites in all PTM data (refer to recall).
