# Supplementary material for: A homology-based pipeline for global prediction of post-translational modification sites
Source: Sci Rep. 2016 May 13;6:25801. doi: 10.1038/srep25801 (PMC4865729; doi:10.1038/srep25801)
Supplement: Supplementary Information [file srep25801-s1.doc]

**Supplemental Information**

**A homology-based pipeline for global prediction of post-translational modification sites**

**Xiang Chen^1^, Shao-Ping Shi^1,2^, Hao-Dong Xu^1^, Sheng-Bao Suo^1^, Jian-Ding Qiu^1,3^***

^1^ Department of Chemistry, Nanchang University, Nanchang 330031, P.R.China

^2^ Department of Mathematics, Nanchang University, Nanchang 330031, P.R.China

^3^ Department of Materials and Chemical Engineering, Pingxiang University, Pingxiang 337055, P.R.China

* To whom correspondence should be addressed. Tel: + 86 791 83969518; Email: jdqiu@ncu.edu.cn

**Supplemental Text 1.**

In this example, we search for the cellular tumor antigen p53 from *Macaca mulatta* as a query and use default options to obtain predictions for phosphorylation. The result page includes two parts: detailed submission form and search results for one search. The search results will be presented as a searchable table in which each row shows a predicted phosphorylation site. In case of p53, we get a list of 32 predictions for 32 phosphorylation sites, and the user can choose view number of the predictions in the table, which default only the first 10 of these predictions are shown in the table (see Supplemental Figure 2D). For each prediction we list nine columns: The first and fourth columns respectively represent the protein name (UniProt ID) for query protein and the protein of known PTM. The second and fifth column respectively indicate the putative and experimentally-verified modification residues as well as its positions in query protein and the protein of known PTM. The third and sixth columns respectively show the putative and experimentally-verified modification residues surrounding sequence segments in query protein and the protein of known PTM. The seventh column represents the number of non-conservative sequence differences between the peptide of known PTM and query peptide. The eighth column represents the expect value of alignment between query peptide and peptide of known PTM. The ninth column represents the status of CPE (cross-promotion expect-value, see method section) aligned between query protein and protein known PTM. It should be noted that the user could determine PTM status by the CPE (yes or no). However, if both sequence differences in the seventh column and E-value in the eighth column should preferably be low, the prediction can also be considered as a candidate of PTM site even in “no” status for CPE. Notably, those predictions are searchable by entering key words to textbox in the table. Furthermore, the user can investigate the predictions in greater detail via the web interface. For each known protein and query protein, we link to UniProt database where the user can find manually curated information on sequence annotation column (see Supplemental Figure 2D).

**Supplemental Figure 1. Identification results of protein Q8SPZ3 from *Delphinapterus leucas* and protein P61260 from *Macaca fuscata* in PTMProber system.** Different colors illustrate exactly identification sites for different PTM types. Phosphorylation, acetylation, ubiquitination, methylation and sumoylation respectively are represented as blue, red, yellow, purple and green.

**
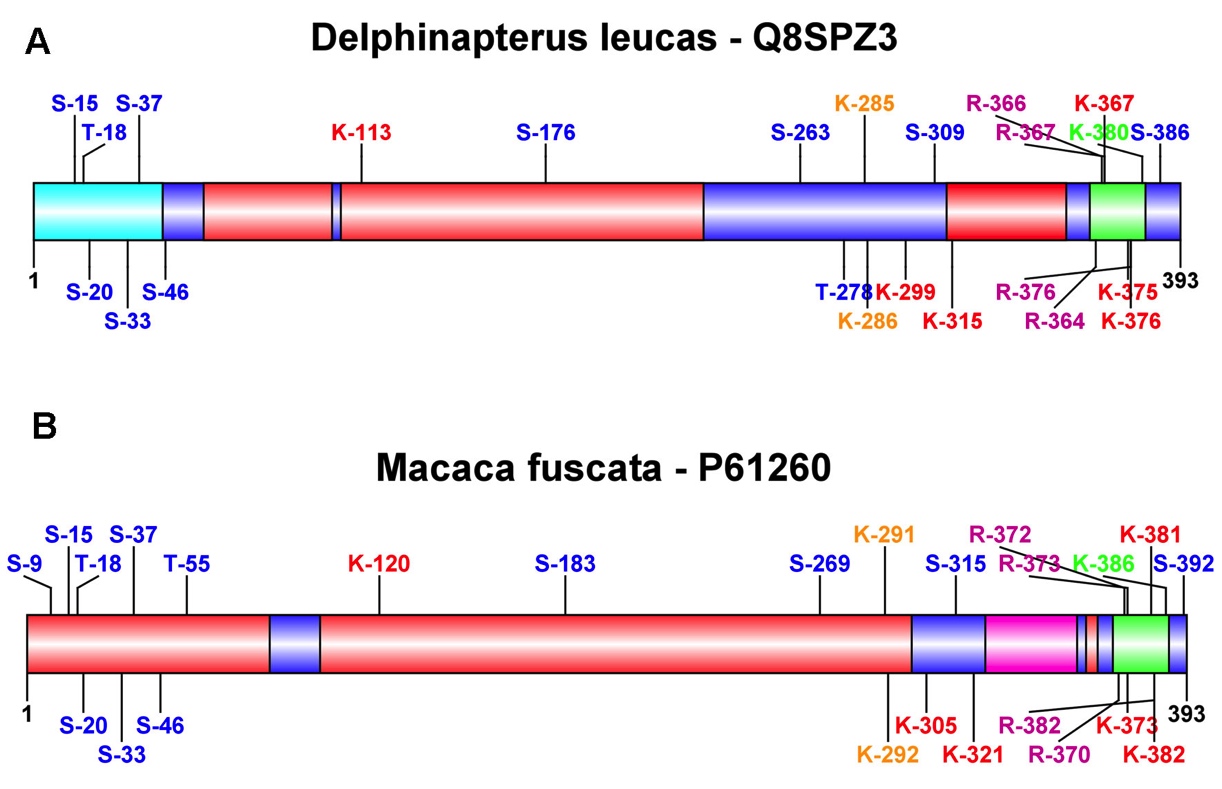
**

**Supplemental Figure 2. Main work interface of PTMProber.** A popular human protein TP53 protein is interested because that is frequently mutated or inactivated in about 60% of cancers. The homologous protein (UniProtID: P56424) in Macaca mulatta (taxid=9544) is chosen as the example protein to search phosphorylation sites in search interface (A). Extensible tool on setting BLAST database of query proteome is used when the database is not in the list (B). Extensible tool on setting BLAST database of known PTM is utilized to customize individual database by user-provided PTM data (C). The system returns user-submitted form and searching results involving a total of 32 phosphorylation sites which are presented in searchable tabular view (only the first 10 predictions are shown), and these results can now be sent to user-provided email address and investigated in further detail by following the links to the UniProt database for curated knowledge related to the sites (D).

**
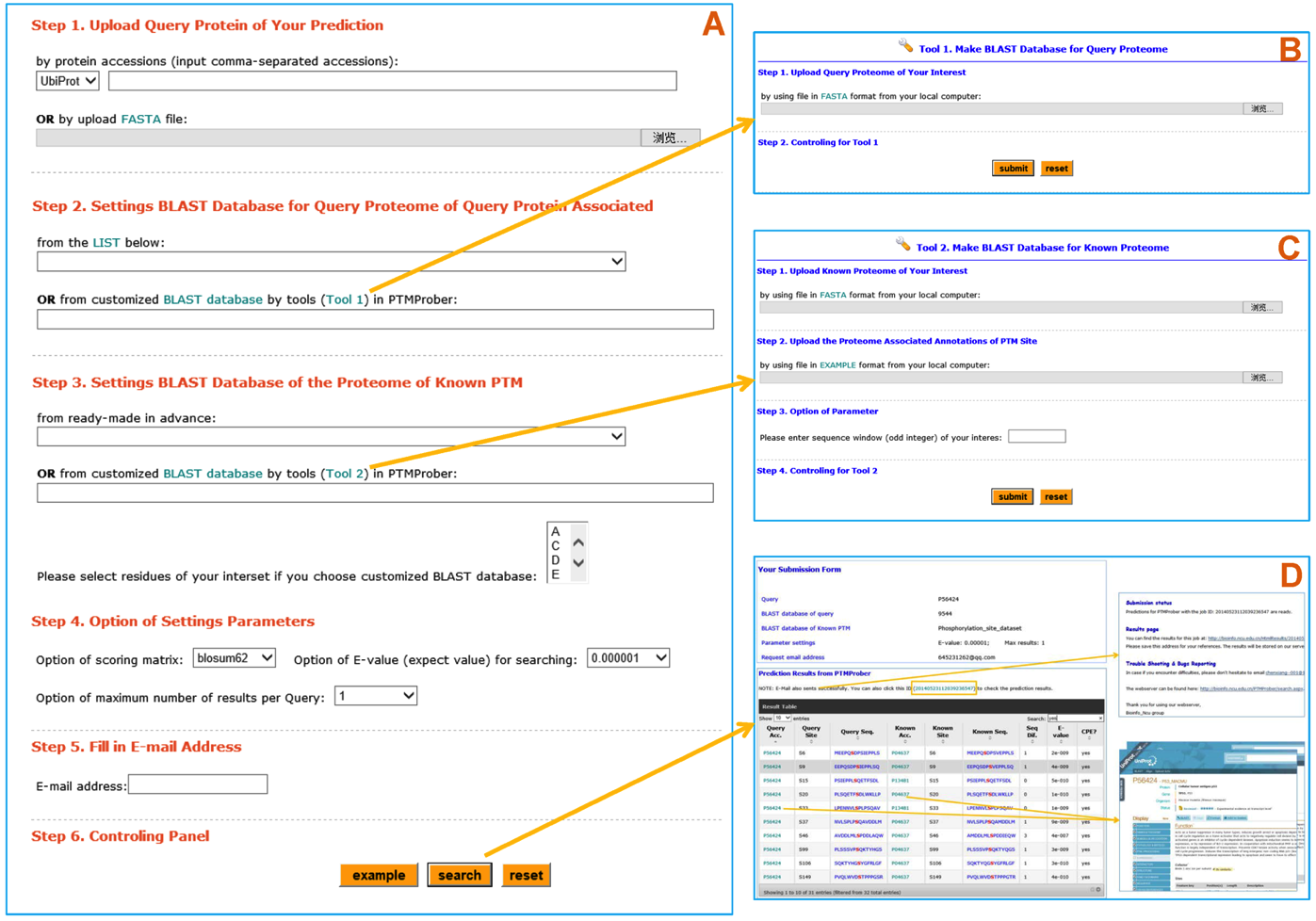
**

**Supplemental Table 1. the list shows the protein PTM data for testing PTMProber including protein and site number (*P. num.* and *S. num.*) in *Rattus norvegicus*, *Gallus gallus* and *Gallus gallus*.**

| ***PTM*** | ***Rattus norvegicus*** | | |  | ***Gallus gallus*** | |  | ***Bos taurus*** | |
| --- | --- | --- | --- | --- | --- | --- | --- | --- | --- |
|  | ***P. num.*** | ***S. num.*** | |  | ***P. num.*** | ***S. num.*** |  | ***P. num.*** | ***S. num.*** |
| *Phosphorylation* | *3248* | | *13863* |  | *69* | *367* |  | *140* | *508* |
| *Acetylation* | *202* | | *620* |  | *5* | *13* |  | *10* | *41* |
| *Ubiquitination* | *511* | | *1889* |  | *1* | *1* |  | *1* | *2* |
| *Sumoylation* | *12* | | *29* |  | *2* | *4* |  | *2* | *5* |
| *Methylation* | *173* | | *505* |  | *5* | *12* |  | *5* | *22* |

**Supplemental Table 2.** **Comparison CPE benchmark with E-value benchmark.** The “Seq. Diff.” column indicates the number of sequence differences between a query site in *testing data* and its best known site match from PhosphoSitePlus/UniProt. The “NH” row indicates query sites for which there was either no known site match in PhosphoSitePlus/UniProt. The “no homology” row in “CPE” column indicates that either the query site had no match in the PhosphoSitePlus/UniProt, or that “CPE = no”. The “no homology” row in “E-value” column indicates that either the match site E-value is smaller than 10^-6^, or also the query site had no match in the PhosphoSitePlus/UniProt. *NH represents no homology.

| ***Seq.Diff.*** | ***Phosphorylation(%)*** | |  | ***Ubiquitination(%)*** | |  | ***Acetylation(%)*** | |  | ***Methylation(%)*** | |  | ***Sumoylation(%)*** | |
| --- | --- | --- | --- | --- | --- | --- | --- | --- | --- | --- | --- | --- | --- | --- |
|  | ***CPE*** | ***E-value*** |  | ***CPE*** | ***E-value*** |  | ***CPE*** | ***E-value*** |  | ***CPE*** | ***E-value*** |  | ***CPE*** | ***E-value*** |
| 0 | 25.35 | 32.64 |  | 29.42 | 32.47 |  | 31.27 | 36.01 |  | 22.89 | 28.74 |  | 20.33 | 24.89 |
| 1 | 13.22 | 20.31 |  | 15.22 | 19.66 |  | 14.63 | 18.39 |  | 11.18 | 15.11 |  | 10.28 | 12.66 |
| 2 | 6.21 | 10.22 |  | 5.29 | 5.89 |  | 8.43 | 10.25 |  | 4.78 | 7.33 |  | 4.21 | 7.76 |
| 3 | 1.25 | 4.77 |  | 2.18 | 3.39 |  | 4.88 | 4.73 |  | 2.42 | 3.86 |  | 2.81 | 4.01 |
| 4 | 0.79 | 4.02 |  | 1.01 | 2.31 |  | 1.74 | 3.20 |  | 1.21 | 2.46 |  | 1.00 | 2.34 |
| 5 | 0.46 | 3.20 |  | 0.68 | 1.39 |  | 1.25 | 1.19 |  | 0.50 | 0.88 |  | 0.37 | 0.85 |
| 6 | 0.31 | 1.76 |  | 0.52 | 1.21 |  | 0.81 | 1.01 |  | 0.25 | 0.51 |  | 0.37 | 0.55 |
| 7 | 0.08 | 0.99 |  | 0.18 | 0.67 |  | 0.72 | 0.45 |  | 0.18 | 0.48 |  | 0.08 | 0.29 |
| 8+ | 0.04 | 0.29 |  | 0.11 | 0.19 |  | 0.02 | 0.26 |  | 0.09 | 0.25 |  | 0.02 | 0.19 |
| *NH | 52.29 | 21.8 |  | 45.39 | 32.82 |  | 36.25 | 24.51 |  | 56.5 | 40.38 |  | 60.53 | 46.46 |

**Supplemental Table 3. PTM data sources and statistics for different PTM types.**

| ***PTM Types*** | ***PTM Residues*** | ***Protein Num.*** | ***Peptide Num.*** | ***Organism Num.*** |
| --- | --- | --- | --- | --- |
| *phosphorylation* | *serine/threonine/tyrosine* | *29275* | *192840* | *23* |
| *acetylation* | *lysine* | *5837* | *15068* | *8* |
| *ubiquitination* | *lysine* | *14090* | *55978* | *6* |
| *methylation* | *lysine/arginine* | *2714* | *6614* | *8* |
| *sumoylation* | *lysine* | *476* | *854* | *5* |

**Supplemental Table 4. Organism index of 340 cellular conditions in PTMProber V1.0.**

| ***Taxonomy ID*** | ***Organism Name*** |
| --- | --- |
| *taxid=2880* | *Ectocarpus siliculosus (Brown alga)* |
| *taxid=3055* | *Chlamydomonas reinhardtii* |
| *taxid=3067* | *Volvox carteri (Green alga)* |
| *taxid=3694* | *Populus trichocarpa (Western balsam poplar)* |
| *taxid=3702* | *Arabidopsis thaliana (Mouse-ear cress)* |
| *taxid=3847* | *Glycine max (Soybean)* |
| *taxid=3988* | *Ricinus communis (Castor bean)* |
| *taxid=4533* | *Oryza brachyantha* |
| *taxid=4538* | *Oryza glaberrima (African rice)* |
| *taxid=4558* | *Sorghum bicolor (Sorghum)* |
| *taxid=5660* | *Leishmania braziliensis* |
| *taxid=5664* | *Leishmania major* |
| *taxid=5671* | *Leishmania infantum* |
| *taxid=5693* | *Trypanosoma cruzi* |
| *taxid=5722* | *Trichomonas vaginalis* |
| *taxid=5759* | *Entamoeba histolytica* |
| *taxid=5762* | *Naegleria gruberi (Amoeba)* |
| *taxid=5786* | *Dictyostelium purpureum (Slime mold)* |
| *taxid=5811* | *Toxoplasma gondii* |
| *taxid=5823* | *Plasmodium berghei (strain Anka)* |
| *taxid=5825* | *Plasmodium chabaudi* |
| *taxid=5851* | *Plasmodium knowlesi (strain H)* |
| *taxid=5865* | *Babesia bovis* |
| *taxid=5874* | *Theileria annulata* |
| *taxid=5875* | *Theileria parva (East coast fever infection agent)* |
| *taxid=5888* | *Paramecium tetraurelia* |
| *taxid=6183* | *Schistosoma mansoni (Blood fluke)* |
| *taxid=6238* | *Caenorhabditis briggsae* |
| *taxid=6239* | *Caenorhabditis elegans* |
| *taxid=6279* | *Brugia malayi (Filarial nematode worm)* |
| *taxid=6293* | *Wuchereria bancrofti* |
| *taxid=6334* | *Trichinella spiralis (Trichina worm)* |
| *taxid=6669* | *Daphnia pulex (Water flea)* |
| *taxid=6945* | *Ixodes scapularis (Black-legged tick)* |
| *taxid=7029* | *Acyrthosiphon pisum (Pea aphid)* |
| *taxid=7070* | *Tribolium castaneum (Red flour beetle)* |
| *taxid=7091* | *Bombyx mori (Silk moth)* |
| *taxid=7159* | *Aedes aegypti (Yellowfever mosquito)* |
| *taxid=7165* | *Anopheles gambiae (African malaria mosquito)* |
| *taxid=7176* | *Culex quinquefasciatus (Southern house mosquito)* |
| *taxid=7209* | *Loa loa (Eye worm)* |
| *taxid=7217* | *Drosophila ananassae (Fruit fly)* |
| *taxid=7220* | *Drosophila erecta (Fruit fly)* |
| *taxid=7222* | *Drosophila grimshawi (Fruit fly)* |
| *taxid=7227* | *Drosophila melanogaster (Fruit fly)* |
| *taxid=7230* | *Drosophila mojavensis (Fruit fly)* |
| *taxid=7234* | *Drosophila persimilis (Fruit fly)* |
| *taxid=7238* | *Drosophila sechellia (Fruit fly)* |
| *taxid=7240* | *Drosophila simulans (Fruit fly)* |
| *taxid=7244* | *Drosophila virilis (Fruit fly)* |
| *taxid=7245* | *Drosophila yakuba (Fruit fly)* |
| *taxid=7260* | *Drosophila willistoni (Fruit fly)* |
| *taxid=7425* | *Nasonia vitripennis (Parasitic wasp)* |
| *taxid=7460* | *Apis mellifera (Honeybee)* |
| *taxid=7668* | *Strongylocentrotus purpuratus (Purple sea urchin)* |
| *taxid=7719* | *Ciona intestinalis (Transparent sea squirt)* |
| *taxid=7739* | *Branchiostoma floridae (Florida lancelet)* |
| *taxid=7897* | *Latimeria chalumnae (West Indian ocean coelacanth)* |
| *taxid=7955* | *Danio rerio (Zebrafish)* |
| *taxid=7998* | *Ictalurus punctatus (Channel catfish) (Silurus punctatus)* |
| *taxid=8022* | *Oncorhynchus mykiss (Rainbow trout) (Salmo gairdneri)* |
| *taxid=8083* | *Xiphophorus maculatus (Southern platyfish) (Platypoecilus maculatus)* |
| *taxid=8084* | *Xiphophorus helleri (Green swordtail)* |
| *taxid=8090* | *Oryzias latipes (Medaka fish)* |
| *taxid=8128* | *Oreochromis niloticus (Nile tilapia)* |
| *taxid=8260* | *Platichthys flesus (European flounder) (Pleuronectes flesus)* |
| *taxid=8364* | *Xenopus tropicalis (Western clawed frog)* |
| *taxid=9031* | *Gallus gallus (Chicken)* |
| *taxid=9103* | *Meleagris gallopavo (Common turkey)* |
| *taxid=9258* | *Ornithorhynchus anatinus (Duckbill platypus)* |
| *taxid=9305* | *Sarcophilus harrisii (Tasmanian devil)* |
| *taxid=9483* | *Callithrix jacchus (White-tufted-ear marmoset)* |
| *taxid=9541* | *Macaca fascicularis (Crab-eating macaque)* |
| *taxid=9543* | *Macaca fuscata fuscata (Japanese macaque)* |
| *taxid=9544* | *Macaca mulatta (Rhesus macaque)* |
| *taxid=9595* | *Gorilla gorilla gorilla (Lowland gorilla)* |
| *taxid=9598* | *Pan troglodytes (Chimpanzee)* |
| *taxid=9601* | *Pongo abelii (Sumatran orangutan)* |
| *taxid=9606* | *Homo sapiens (Human)* |
| *taxid=9615* | *Canis familiaris (Dog)* |
| *taxid=9646* | *Ailuropoda melanoleuca (Giant panda)* |
| *taxid=9749* | *Delphinapterus leucas (Beluga whale)* |
| *taxid=9785* | *Loxodonta africana (African elephant)* |
| *taxid=9796* | *Equus caballus (Horse)* |
| *taxid=9823* | *Sus scrofa (Pig)* |
| *taxid=9913* | *Bos taurus (Bovine)* |
| *taxid=9986* | *Oryctolagus cuniculus (Rabbit)* |
| *taxid=10029* | *Cricetulus griseus (Chinese hamster)* |
| *taxid=10090* | *Mus musculus (Mouse)* |
| *taxid=10116* | *Rattus norvegicus (Rat)* |
| *taxid=10141* | *Cavia porcellus (Guinea pig)* |
| *taxid=10181* | *Heterocephalus glaber (Naked mole rat)* |
| *taxid=10228* | *Trichoplax adhaerens* |
| *taxid=12957* | *Atta cephalotes (Leafcutter ant)* |
| *taxid=12968* | *Blastocystis hominis* |
| *taxid=13037* | *Danaus plexippus (Monarch butterfly)* |
| *taxid=13616* | *Monodelphis domestica (Gray short-tailed opossum)* |
| *taxid=13642* | *Polysphondylium pallidum (Cellular slime mold)* |
| *taxid=13686* | *Solenopsis invicta (Red imported fire ant)* |
| *taxid=15368* | *Brachypodium distachyon (Purple false brome)* |
| *taxid=28377* | *Anolis carolinensis (Green anole)* |
| *taxid=29159* | *Crassostrea gigas (Pacific oyster)* |
| *taxid=29760* | *Vitis vinifera (Grape)* |
| *taxid=30611* | *Otolemur garnettii (Small-eared galago)* |
| *taxid=31033* | *Takifugu rubripes (Japanese pufferfish)* |
| *taxid=31234* | *Caenorhabditis remanei* |
| *taxid=34765* | *Oikopleura dioica (Tunicate)* |
| *taxid=35128* | *Thalassiosira pseudonana (Marine diatom)* |
| *taxid=36329* | *Plasmodium falciparum (isolate 3D7)* |
| *taxid=39947* | *Oryza sativa subsp. japonica (Rice)* |
| *taxid=40830* | *Barbus barbus (Barbel) (Cyprinus barbus)* |
| *taxid=43151* | *Anopheles darlingi (Mosquito)* |
| *taxid=43179* | *Spermophilus tridecemlineatus (Thirteen-lined ground squirrel)* |
| *taxid=44056* | *Aureococcus anophagefferens (Harmful bloom alga)* |
| *taxid=44689* | *Dictyostelium discoideum (Slime mold)* |
| *taxid=45351* | *Nematostella vectensis (Starlet sea anemone)* |
| *taxid=46245* | *Drosophila pseudoobscura pseudoobscura (Fruit fly)* |
| *taxid=51511* | *Ciona savignyi (Pacific transparent sea squirt)* |
| *taxid=54126* | *Pristionchus pacificus* |
| *taxid=59463* | *Myotis lucifugus (Little brown bat)* |
| *taxid=59729* | *Taeniopygia guttata (Zebra finch)* |
| *taxid=61853* | *Nomascus leucogenys (Northern white-cheeked gibbon)* |
| *taxid=69293* | *Gasterosteus aculeatus (Three-spined stickleback)* |
| *taxid=70448* | *Ostreococcus tauri* |
| *taxid=73239* | *Plasmodium yoelii yoelii* |
| *taxid=79923* | *Clonorchis sinensis (Chinese liver fluke)* |
| *taxid=81824* | *Monosiga brevicollis (Choanoflagellate)* |
| *taxid=81972* | *Arabidopsis lyrata subsp. lyrata (Lyre-leaved rock-cress)* |
| *taxid=88036* | *Selaginella moellendorffii (Spikemoss)* |
| *taxid=94908* | *Tetraodon miurus (Congo puffer)* |
| *taxid=99883* | *Tetraodon nigroviridis (Spotted green pufferfish)* |
| *taxid=103372* | *Acromyrmex echinatior (Panamanian leafcutter ant)* |
| *taxid=104421* | *Camponotus floridanus (Florida carpenter ant)* |
| *taxid=121224* | *Pediculus humanus subsp. corporis (Body louse)* |
| *taxid=126793* | *Plasmodium vivax (strain Salvador I)* |
| *taxid=135651* | *Caenorhabditis brenneri (Nematode worm)* |
| *taxid=145481* | *Physcomitrella patens subsp. patens (Moss)* |
| *taxid=159749* | *Thalassiosira oceanica (Marine diatom)* |
| *taxid=164328* | *Phytophthora ramorum (Sudden oak death agent)* |
| *taxid=184922* | *Giardia intestinalis (strain ATCC 50803 / WB clone C6)* |
| *taxid=214684* | *Cryptococcus neoformans var. neoformans serotype D (strain JEC21 / ATCC MYA-565)* |
| *taxid=222929* | *Coccidioides posadasii (strain C735) (Valley fever fungus)* |
| *taxid=226230* | *Saccharomyces kudriavzevii (strain ATCC MYA-4449 / AS 2.2408 / CBS 8840 / NBRC 1802 / NCYC 2889)* |
| *taxid=227321* | *Emericella nidulans (strain FGSC A4 / ATCC 38163 / CBS 112.46 / NRRL 194 / M139)* |
| *taxid=229533* | *Gibberella zeae (strain PH-1 / ATCC MYA-4620 / FGSC 9075 / NRRL 31084) (Wheat head blight fungus)* |
| *taxid=235443* | *Cryptococcus neoformans var. grubii serotype A (strain H99 / ATCC 208821 / CBS 10515 / FGSC 9487)* |
| *taxid=237561* | *Candida albicans (strain SC5314 / ATCC MYA-2876) (Yeast)* |
| *taxid=237631* | *Ustilago maydis (strain 521 / FGSC 9021) (Corn smut fungus)* |
| *taxid=237895* | *Cryptosporidium hominis* |
| *taxid=240176* | *Coprinopsis cinerea (strain Okayama-7 / 130 / ATCC MYA-4618 / FGSC 9003) (Inky cap fungus)* |
| *taxid=242507* | *Magnaporthe oryzae (strain 70-15 / ATCC MYA-4617 / FGSC 8958) (Rice blast fungus)* |
| *taxid=246409* | *Rhizopus delemar (strain RA 99-880 / ATCC MYA-4621 / FGSC 9543 / NRRL 43880) (Mucormycosis agent)* |
| *taxid=246410* | *Coccidioides immitis (strain RS) (Valley fever fungus)* |
| *taxid=246437* | *Tupaia chinensis (Chinese tree shrew)* |
| *taxid=281687* | *Caenorhabditis japonica* |
| *taxid=283643* | *Cryptococcus neoformans var. neoformans serotype D (strain B-3501A)* |
| *taxid=284590* | *Kluyveromyces lactis (strain ATCC 8585 / CBS 2359 / DSM 70799 / NBRC 1267 / NRRL Y-1140 / WM37) (Yeast)* |
| *taxid=284591* | *Yarrowia lipolytica (strain CLIB 122 / E 150) (Yeast)* |
| *taxid=284592* | *Debaryomyces hansenii (strain ATCC 36239 / CBS 767 / JCM 1990 / NBRC 0083 / IGC 2968) (Yeast)* |
| *taxid=284593* | *Candida glabrata (strain ATCC 2001 / CBS 138 / JCM 3761 / NBRC 0622 / NRRL Y-65) (Yeast)* |
| *taxid=284811* | *Ashbya gossypii (strain ATCC 10895 / CBS 109.51 / FGSC 9923 / NRRL Y-1056) (Yeast)* |
| *taxid=284812* | *Schizosaccharomyces pombe (strain 972 / ATCC 24843) (Fission yeast)* |
| *taxid=284813* | *Encephalitozoon cuniculi (strain GB-M1) (Microsporidian parasite)* |
| *taxid=285006* | *Saccharomyces cerevisiae (strain RM11-1a) (Baker's yeast)* |
| *taxid=294746* | *Meyerozyma guilliermondii (strain ATCC 6260 / CBS 566 / DSM 6381 / JCM 1539 / NBRC 10279 / NRRL Y-324) (Yeast)* |
| *taxid=294747* | *Candida tropicalis (strain ATCC MYA-3404 / T1) (Yeast)* |
| *taxid=294748* | *Candida albicans (strain WO-1) (Yeast)* |
| *taxid=296587* | *Micromonas sp. (strain RCC299 / NOUM17) (Picoplanktonic green alga)* |
| *taxid=306901* | *Chaetomium globosum (strain ATCC 6205 / CBS 148.51 / DSM 1962 / NBRC 6347 / NRRL 1970) (Soil fungus)* |
| *taxid=306902* | *Clavispora lusitaniae (strain ATCC 42720) (Yeast)* |
| *taxid=307796* | *Saccharomyces cerevisiae (strain YJM789) (Baker's yeast)* |
| *taxid=312017* | *Tetrahymena thermophila (strain SB210)* |
| *taxid=321614* | *Phaeosphaeria nodorum (strain SN15 / ATCC MYA-4574 / FGSC 10173) (Glume blotch fungus)* |
| *taxid=322104* | *Scheffersomyces stipitis (strain ATCC 58785 / CBS 6054 / NBRC 10063 / NRRL Y-11545) (Yeast)* |
| *taxid=330879* | *Neosartorya fumigata (strain ATCC MYA-4609 / Af293 / CBS 101355 / FGSC A1100)* |
| *taxid=331117* | *Neosartorya fischeri (strain ATCC 1020 / DSM 3700 / FGSC A1164 / NRRL 181)* |
| *taxid=332952* | *Aspergillus flavus (strain ATCC 200026 / FGSC A1120 / NRRL 3357 / JCM 12722 / SRRC 167)* |
| *taxid=336722* | *Mycosphaerella graminicola (strain CBS 115943 / IPO323) (Speckled leaf blotch fungus)* |
| *taxid=336963* | *Uncinocarpus reesii (strain UAMH 1704)* |
| *taxid=339724* | *Ajellomyces capsulata (strain NAm1 / WU24) (Darling's disease fungus)* |
| *taxid=341663* | *Aspergillus terreus (strain NIH 2624 / FGSC A1156)* |
| *taxid=344612* | *Aspergillus clavatus (strain ATCC 1007 / CBS 513.65 / DSM 816 / NCTC 3887 / NRRL 1)* |
| *taxid=353152* | *Cryptosporidium parvum (strain Iowa II)* |
| *taxid=353153* | *Trypanosoma cruzi (strain CL Brener)* |
| *taxid=367110* | *Neurospora crassa (strain ATCC 24698 / 74-OR23-1A / CBS 708.71 / DSM 1257 / FGSC 987)* |
| *taxid=367775* | *Cryptococcus gattii serotype B (strain WM276 / ATCC MYA-4071) (Filobasidiella gattii)* |
| *taxid=370354* | *Entamoeba dispar (strain ATCC PRA-260 / SAW760)* |
| *taxid=379508* | *Lodderomyces elongisporus (strain ATCC 11503 / CBS 2605 / JCM 1781 / NBRC 1676 / NRRL YB-4239) (Yeast)* |
| *taxid=380704* | *Aspergillus niger (strain ATCC 1015 / CBS 113.46 / FGSC A1144 / LSHB Ac4 / NCTC 3858a / NRRL 328 / USDA 3528.7)* |
| *taxid=400682* | *Amphimedon queenslandica (Sponge)* |
| *taxid=402676* | *Schizosaccharomyces japonicus (strain yFS275 / FY16936) (Fission yeast)* |
| *taxid=403677* | *Phytophthora infestans (strain T30-4) (Potato late blight fungus)* |
| *taxid=413071* | *Hypocrea virens (strain Gv29-8 / FGSC 10586) (Gliocladium virens)* |
| *taxid=418459* | *Puccinia graminis f. sp. tritici (strain CRL 75-36-700-3 / race SCCL) (Black stem rust fungus)* |
| *taxid=423536* | *Perkinsus marinus (strain ATCC 50983 / TXsc)* |
| *taxid=425011* | *Aspergillus niger (strain CBS 513.88 / FGSC A1513)* |
| *taxid=425265* | *Malassezia globosa (strain ATCC MYA-4612 / CBS 7966) (Dandruff-associated fungus)* |
| *taxid=426418* | *Pyrenophora tritici-repentis (strain Pt-1C-BFP) (Wheat tan spot fungus)* |
| *taxid=426428* | *Fusarium oxysporum f. sp. lycopersici (strain 4287 / CBS 123668 / FGSC 9935 / NRRL 34936) (Fusarium vascular wilt of tomato)* |
| *taxid=431241* | *Hypocrea jecorina (strain QM6a)* |
| *taxid=436017* | *Ostreococcus lucimarinus (strain CCE9901)* |
| *taxid=436907* | *Vanderwaltozyma polyspora (strain ATCC 22028 / DSM 70294)* |
| *taxid=441375* | *Cryptosporidium muris (strain RN66)* |
| *taxid=441959* | *Talaromyces stipitatus (strain ATCC 10500 / CBS 375.48 / QM 6759 / NRRL 1006)* |
| *taxid=441960* | *Penicillium marneffei (strain ATCC 18224 / CBS 334.59 / QM 7333)* |
| *taxid=443226* | *Coccidioides posadasii (strain RMSCC 757 / Silveira) (Valley fever fungus)* |
| *taxid=447093* | *Ajellomyces capsulata (strain G186AR / H82 / ATCC MYA-2454 / RMSCC 2432) (Darling's disease fungus)* |
| *taxid=451804* | *Neosartorya fumigata (strain CEA10 / CBS 144.89 / FGSC A1163)* |
| *taxid=452589* | *Hypocrea atroviridis (strain ATCC 20476 / IMI 206040)* |
| *taxid=481877* | *Enterocytozoon bieneusi (strain H348) (Microsporidian parasite)* |
| *taxid=482561* | *Paracoccidioides brasiliensis (strain Pb03)* |
| *taxid=486041* | *Laccaria bicolor (strain S238N-H82 / ATCC MYA-4686) (Bicoloured deceiver)* |
| *taxid=498257* | *Verticillium dahliae (strain VdLs.17 / ATCC MYA-4575 / FGSC 10137)* |
| *taxid=500485* | *Penicillium chrysogenum (strain ATCC 28089 / DSM 1075 / Wisconsin 54-1255)* |
| *taxid=502779* | *Paracoccidioides brasiliensis (strain ATCC MYA-826 / Pb01)* |
| *taxid=502780* | *Paracoccidioides brasiliensis (strain Pb18)* |
| *taxid=510516* | *Aspergillus oryzae (strain ATCC 42149 / RIB 40) (Yellow koji mold)* |
| *taxid=510951* | *Neurospora tetrasperma (strain FGSC 2508 / ATCC MYA-4615 / P0657)* |
| *taxid=510952* | *Neurospora tetrasperma (strain FGSC 2509 / P0656)* |
| *taxid=515849* | *Podospora anserina (strain S / ATCC MYA-4624 / DSM 980 / FGSC 10383) (Pleurage anserina)* |
| *taxid=526221* | *Verticillium albo-atrum (strain VaMs.102 / ATCC MYA-4576 / FGSC 10136) (Verticillium wilt)* |
| *taxid=535722* | *Arthroderma gypseum (strain ATCC MYA-4604 / CBS 118893)* |
| *taxid=544711* | *Ajellomyces capsulata (strain H88) (Darling's disease fungus)* |
| *taxid=544712* | *Ajellomyces capsulata (strain H143) (Darling's disease fungus)* |
| *taxid=545124* | *Saccharomyces cerevisiae (strain AWRI1631) (Baker's yeast)* |
| *taxid=554065* | *Chlorella variabilis (Green alga)* |
| *taxid=554155* | *Arthroderma otae (strain ATCC MYA-4605 / CBS 113480)* |
| *taxid=554373* | *Moniliophthora perniciosa (strain FA553 / isolate CP02) (Witches'-broom disease fungus)* |
| *taxid=556484* | *Phaeodactylum tricornutum (strain CCAP 1055/1)* |
| *taxid=559292* | *Saccharomyces cerevisiae (strain ATCC 204508 / S288c) (Baker's yeast)* |
| *taxid=559295* | *Lachancea thermotolerans (strain ATCC 56472 / CBS 6340 / NRRL Y-8284) (Yeast)* |
| *taxid=559297* | *Ajellomyces dermatitidis (strain ER-3 / ATCC MYA-2586)* |
| *taxid=559298* | *Ajellomyces dermatitidis (strain SLH14081)* |
| *taxid=559304* | *Pichia sorbitophila (strain ATCC MYA-4447 / BCRC 22081 / CBS 7064 / NBRC 10061 / NRRL Y-12695) (Hybrid yeast)* |
| *taxid=559305* | *Trichophyton rubrum (strain ATCC MYA-4607 / CBS 118892) (Athlete's foot fungus)* |
| *taxid=559307* | *Zygosaccharomyces rouxii (strain ATCC 2623 / CBS 732 / NBRC 1130 / NCYC 568 / NRRL Y-229)* |
| *taxid=559882* | *Trichophyton equinum (strain ATCC MYA-4606 / CBS 127.97) (Horse ringworm fungus)* |
| *taxid=561896* | *Postia placenta (strain ATCC 44394 / Madison 698-R) (Brown rot fungus)* |
| *taxid=564608* | *Micromonas pusilla (strain CCMP1545) (Picoplanktonic green alga)* |
| *taxid=572307* | *Neospora caninum (strain Liverpool)* |
| *taxid=573729* | *Thielavia heterothallica (strain ATCC 42464 / BCRC 31852 / DSM 1799)* |
| *taxid=573826* | *Candida dubliniensis (strain CD36 / ATCC MYA-646 / CBS 7987 / NCPF 3949 / NRRL Y-17841) (Yeast)* |
| *taxid=574961* | *Saccharomyces cerevisiae (strain JAY291) (Baker's yeast)* |
| *taxid=578454* | *Candida parapsilosis (strain CDC 317 / ATCC MYA-4646) (Yeast)* |
| *taxid=578455* | *Thielavia terrestris (strain ATCC 38088 / NRRL 8126)* |
| *taxid=578457* | *Serpula lacrymans var. lacrymans (strain S7.9) (Dry rot fungus)* |
| *taxid=578458* | *Schizophyllum commune (strain H4-8 / FGSC 9210) (Split gill fungus)* |
| *taxid=578460* | *Nosema ceranae (strain BRL01) (Microsporidian parasite)* |
| *taxid=590646* | *Candida tenuis (strain ATCC 10573 / BCRC 21748 / CBS 615 / JCM 9827 / NBRC 10315 / NRRL Y-1498 / VKM Y-70) (Yeast)* |
| *taxid=595528* | *Capsaspora owczarzaki (strain ATCC 30864)* |
| *taxid=597362* | *Agaricus bisporus var. burnettii (strain JB137-S8 / ATCC MYA-4627 / FGSC 10392) (White button mushroom)* |
| *taxid=598745* | *Giardia intestinalis (strain ATCC 50581 / GS clone H7)* |
| *taxid=610380* | *Harpegnathos saltator (Jerdon's jumping ant)* |
| *taxid=619300* | *Spathaspora passalidarum (strain NRRL Y-27907 / 11-Y1)* |
| *taxid=630390* | *Puccinia triticina (isolate 1-1 / race 1 (BBBD)) (Brown leaf rust fungus)* |
| *taxid=643680* | *Saccharomyces cerevisiae (strain Lalvin EC1118 / Prise de mousse) (Baker's yeast)* |
| *taxid=644223* | *Komagataella pastoris (strain GS115 / ATCC 20864) (Yeast)* |
| *taxid=644352* | *Gaeumannomyces graminis var. tritici (strain R3-111a-1) (Wheat and barley take-all root rot fungus)* |
| *taxid=645133* | *Colletotrichum graminicola (strain M1.001 / M2 / FGSC 10212) (Maize anthracnose fungus)* |
| *taxid=647933* | *Trichophyton tonsurans (strain CBS 112818) (Scalp ringworm fungus)* |
| *taxid=650164* | *Phanerochaete carnosa (strain HHB-10118-sp) (White-rot fungus)* |
| *taxid=653446* | *Ajellomyces dermatitidis (strain ATCC 18188 / CBS 674.68)* |
| *taxid=655819* | *Beauveria bassiana (strain ARSEF 2860) (White muscardine disease fungus)* |
| *taxid=655827* | *Metarhizium acridum (strain CQMa 102)* |
| *taxid=655844* | *Metarhizium anisopliae (strain ARSEF 23 / ATCC MYA-3075)* |
| *taxid=655863* | *Grosmannia clavigera (strain kw1407 / UAMH 11150) (Blue stain fungus)* |
| *taxid=656061* | *Tuber melanosporum (strain Mel28) (Perigord black truffle)* |
| *taxid=658858* | *Giardia intestinalis (strain P15)* |
| *taxid=660025* | *Fusarium oxysporum (strain Fo5176) (Panama disease fungus)* |
| *taxid=660122* | *Nectria haematococca (strain 77-13-4 / ATCC MYA-4622 / FGSC 9596 / MPVI)* |
| *taxid=663202* | *Trichophyton verrucosum (strain HKI 0517)* |
| *taxid=663331* | *Arthroderma benhamiae (strain ATCC MYA-4681 / CBS 112371)* |
| *taxid=665079* | *Sclerotinia sclerotiorum (strain ATCC 18683 / 1980 / Ss-1) (White mold)* |
| *taxid=671144* | *Wallemia sebi (strain ATCC MYA-4683 / CBS 633.66)* |
| *taxid=679716* | *Trypanosoma brucei gambiense (strain MHOM/CI/86/DAL972)* |
| *taxid=684364* | *Batrachochytrium dendrobatidis (strain JAM81 / FGSC 10211) (Frog chytrid fungus)* |
| *taxid=717982* | *Auricularia delicata (strain TFB10046) (White-rot fungus)* |
| *taxid=721032* | *Saccharomyces cerevisiae (strain Kyokai no. 7 / NBRC 101557) (Baker's yeast)* |
| *taxid=747676* | *Melampsora larici-populina (strain 98AG31 / pathotype 3-4-7) (Poplar leaf rust fungus)* |
| *taxid=756982* | *Arthrobotrys oligospora (strain ATCC 24927 / CBS 115.81 / DSM 1491) (Nematode-trapping fungus)* |
| *taxid=759272* | *Chaetomium thermophilum (strain DSM 1495 / CBS 144.50 / IMI 039719)* |
| *taxid=759273* | *Colletotrichum higginsianum (strain IMI 349063) (Crucifer anthracnose fungus)* |
| *taxid=764097* | *Saccharomyces cerevisiae (strain AWRI796) (Baker's yeast)* |
| *taxid=764098* | *Saccharomyces cerevisiae (strain Lalvin QA23) (Baker's yeast)* |
| *taxid=764099* | *Saccharomyces cerevisiae (strain VIN 13) (Baker's yeast)* |
| *taxid=764100* | *Saccharomyces cerevisiae (strain Zymaflore VL3) (Baker's yeast)* |
| *taxid=764101* | *Saccharomyces cerevisiae (strain FostersO) (Baker's yeast)* |
| *taxid=764102* | *Saccharomyces cerevisiae (strain FostersB) (Baker's yeast)* |
| *taxid=764103* | *Mixia osmundae (strain CBS 9802 / IAM 14324 / JCM 22182 / KY 12970)* |
| *taxid=771870* | *Sordaria macrospora (strain ATCC MYA-333 / DSM 997 / K(L3346) / K-hell)* |
| *taxid=857967* | *Ichthyophthirius multifiliis (strain G5) (White spot disease agent)* |
| *taxid=858893* | *Exophiala dermatitidis (strain ATCC 34100 / CBS 525.76 / NIH/UT8656) (Black yeast)* |
| *taxid=861557* | *Pyrenophora teres f. teres (strain 0-1) (Barley net blotch fungus)* |
| *taxid=871575* | *Pichia angusta (strain ATCC 26012 / NRRL Y-7560 / DL-1) (Yeast)* |
| *taxid=876142* | *Encephalitozoon intestinalis (strain ATCC 50506) (Microsporidian parasite)* |
| *taxid=881290* | *Nematocida parisii (strain ERTm1 / ATCC PRA-289) (Nematode killer fungus)* |
| *taxid=907965* | *Encephalitozoon hellem (strain ATCC 50504) (Microsporidian parasite)* |
| *taxid=929439* | *Leishmania mexicana (strain MHOM/GT/2001/U1103)* |
| *taxid=931890* | *Eremothecium cymbalariae (strain CBS 270.75 / DBVPG 7215 / KCTC 17166 / NRRL Y-17582) (Yeast)* |
| *taxid=935791* | *Nematocida parisii (strain ERTm3) (Nematode killer fungus)* |
| *taxid=936435* | *Serpula lacrymans var. lacrymans (strain S7.3) (Dry rot fungus)* |
| *taxid=944018* | *Nematocida sp. 1 (strain ERTm2 / ATCC PRA-371) (Nematode killer fungus)* |
| *taxid=946362* | *Salpingoeca sp. (strain ATCC 50818)* |
| *taxid=981087* | *Leishmania donovani (strain BPK282A1)* |
| *taxid=981350* | *Komagataella pastoris (strain ATCC 76273 / CBS 7435 / CECT 11047 / NRRL Y-11430 / Wegner 21-1) (Yeast)* |
| *taxid=983644* | *Cordyceps militaris (strain CM01) (Caterpillar fungus)* |
| *taxid=985895* | *Leptosphaeria maculans (strain JN3 / isolate v23.1.3 / race Av1-4-5-6-7-8) (Blackleg fungus)* |
| *taxid=999809* | *Sporisorium reilianum (strain SRZ2) (Maize head smut fungus)* |
| *taxid=999810* | *Botryotinia fuckeliana (strain T4) (Noble rot fungus)* |
| *taxid=999953* | *Trypanosoma brucei brucei (strain 927/4 GUTat10.1)* |
| *taxid=1001064* | *Rhodotorula glutinis (strain ATCC 204091 / IIP 30 / MTCC 1151) (Yeast)* |
| *taxid=1003232* | *Edhazardia aedis (strain USNM 41457) (Microsporidian parasite)* |
| *taxid=1028729* | *Fusarium pseudograminearum (strain CS3096) (Wheat and barley crown-rot fungus)* |
| *taxid=1033177* | *Aspergillus kawachii (strain NBRC 4308) (White koji mold)* |
| *taxid=1054147* | *Dictyostelium fasciculatum (strain SH3) (Slime mold)* |
| *taxid=1055687* | *Trypanosoma vivax (strain Y486)* |
| *taxid=1064592* | *Naumovozyma castellii (strain ATCC 76901 / CBS 4309 / NBRC 1992 / NRRL Y-12630) (Yeast)* |
| *taxid=1068625* | *Trypanosoma congolense (strain IL3000)* |
| *taxid=1071378* | *Naumovozyma dairenensis (strain ATCC 10597 / BCRC 20456 / CBS 421 / NBRC 0211 / NRRL Y-12639)* |
| *taxid=1071380* | *Tetrapisispora blattae (strain ATCC 34711 / CBS 6284 / DSM 70876 / NBRC 10599 / NRRL Y-10934 / UCD 77-7) (Yeast)* |
| *taxid=1071381* | *Tetrapisispora phaffii (strain ATCC 24235 / CBS 4417 / NBRC 1672 / NRRL Y-8282 / UCD 70-5) (Yeast)* |
| *taxid=1071382* | *Kazachstania africana (strain ATCC 22294 / BCRC 22015 / CBS 2517 / CECT 1963 / NBRC 1671 / NRRL Y-8276) (Yeast)* |
| *taxid=1071383* | *Kazachstania naganishii (strain ATCC MYA-139 / BCRC 22969 / CBS 8797 / CCRC 22969 / KCTC 17520 / NBRC 10181 / NCYC 3082) (Yeast)* |
| *taxid=1072389* | *Marssonina brunnea f. sp. multigermtubi (strain MB_m1) (Marssonina leaf spot fungus)* |
| *taxid=1076696* | *Entamoeba nuttalli (strain P19) (Amoeba)* |
| *taxid=1076872* | *Torulaspora delbrueckii (strain ATCC 10662 / CBS 1146 / NBRC 0425 / NCYC 2629 / NRRL Y-866) (Yeast)* |
| *taxid=1094619* | *Phytophthora sojae (strain P6497) (Soybean stem and root rot agent)* |
| *taxid=1104152* | *Glarea lozoyensis (strain ATCC 74030 / MF5533)* |
| *taxid=1109443* | *Piriformospora indica (strain DSM 11827)* |
| *taxid=1126212* | *Macrophomina phaseolina (strain MS6) (Charcoal rot fungus)* |
| *taxid=1136231* | *Candida orthopsilosis (strain 90-125) (Yeast)* |
| *taxid=1160506* | *Aspergillus oryzae (strain 3.042) (Yellow koji mold)* |
| *taxid=1160507* | *Saccharomyces arboricola (strain H-6 / AS 2.3317 / CBS 10644) (Yeast)* |
| *taxid=1170229* | *Penicillium digitatum (strain PHI26 / CECT 20796) (Green mold)* |
| *taxid=1170230* | *Penicillium digitatum (strain Pd1 / CECT 20795) (Green mold)* |
| *taxid=1178016* | *Encephalitozoon romaleae (strain SJ-2008) (Microsporidian parasite)* |
| *taxid=1186058* | *Trichosporon asahii var. asahii (strain ATCC 90039 / CBS 2479 / JCM 2466 / KCTC 7840 / NCYC 2677 / UAMH 7654) (Yeast)* |
| *taxid=1206466* | *Wickerhamomyces ciferrii (strain F-60-10 / ATCC 14091 / CBS 111 / JCM 3599 / NBRC 0793 / NRRL Y-1031) (Yeast)* |
| *taxid=1220162* | *Trichosporon asahii var. asahii (strain CBS 8904) (Yeast)* |
| *taxid=2880* | *Ectocarpus siliculosus (Brown alga)* |
